# CO oxidation and organic dyes degradation over graphene–Cu and graphene–CuNi catalysts obtained by solution combustion synthesis

**DOI:** 10.1038/s41598-020-72872-0

**Published:** 2020-09-30

**Authors:** Alexander Khort, Valentin Romanovski, Denis Leybo, Dmitry Moskovskikh

**Affiliations:** 1 KTH Royal Institute of Technology, 10044 Stockholm, Sweden; 2 National University of Science and Technology “MISIS”, Moscow, Russia 119049; 3 Institute of General and Inorganic Chemistry, National Academy of Sciences of Belarus, 220072 Minsk, Belarus

**Keywords:** Catalyst synthesis, Heterogeneous catalysis, Photocatalysis, Graphene, Nanoparticles, Solid-state chemistry

## Abstract

Graphene and its analogs in combination with metal nanopowders are among the most promising catalysts for various industry valuable processes. The newly obtained solution combustion synthesized graphene–Cu and graphene–CuNi nanocomposites were examined in heterogeneous catalysis of thermal activated CO oxidation and photoactivated degradation of acid telon blue and direct blue dyes. The nanocomposites are characterized by a closely connected solution combustion synthesized graphene-metal structure with a number of graphene layers from 1 to 3 and fine metal grains sizes of 31 nm (Cu) and 14 nm (CuNi). The experimental data showed the obtained graphene-metal nanocomposites are among the most effective catalysts for CO oxidation with a temperature of 100% conversion of 150 °C and 200 °C for Cu and CuNi containing catalysts, respectively. At the same time, both nanopowders were found inactive for dyes degradation.

## Introduction

Nanomaterials (NMs) have got a lot of attention among researchers and in the industry due to their superior, and, sometimes, unique functional characteristics in comparison with bulk analogs. Research and industrial areas, associated with surface-related properties, like sensors^1^ and catalysis^2^, are among the most promising for the application of NMs. Such an interest is caused by features of NMs’ structure and morphology, where at least half atoms of an individual particle are on or near the surface, ready for adsorption and interaction with outer molecules. There is no surprise that various NMs are widely used for heterogeneous catalysts in gas conversion processes^3–5^ and water purification^6–8^. In these areas, the research attention is significantly moving from traditionally effective but expensive noble metal-based NM catalysts like Au, Pt, and Pd to new compounds and comprehensive multi-component structures, containing cheaper 3d elements like Cu, Ni and their combinations^9,10^.

One of the main criteria for the effective heterogeneous catalyst is a high value of a specific surface area required for catalytic interaction. That’s why metal and oxide doped graphene (G) and graphene-based (G oxide (GO) and reduced GO) NMs are among the most popular catalysts^11,12^. Moreover, it was shown, for instance, catalysts containing the same oxide dopant in combination with the highly defected structure of GO gives better results for catalytic CO oxidation in comparison with other doped forms of G^13^. Structural defects, presence of functional surface groups, and features of interaction with metal and oxide NPs are considered as playing one of the major roles in catalysis mechanisms.

Recently, composite nanopowders, consistent from newly developed G-type nanostructure, named scsG, on Cu and CuNi nanograins, were obtained by one-step solution combustion synthesis (SCS)^14^. Using the high-intensive SCS reaction allowed to combine the formation of Cu and CuNi nanograins, which are known to be among the best catalysts for controllable G growth^15^, with simultaneous catalytic activation of scsG formation. The obtained scsG-metal NMs have already shown superior gas sensing results and unusual magnetic properties due to the highly defected structure of scsG and close connection with metal grains through transition layers^14^. These features of the newly obtained scsG-metal structures made them promising candidates for application in heterogeneous catalysis processes, among others.

In this article, we present the results of the study of catalytic activity (catalytic CO oxidation and photocatalytic degradation of acid telon blue (ATB) and direct blue (DB) dyes) of scsG-Cu (G@Cu) and scsG-CuNi (G@CuNi) composite nanopowders, obtained by SCS approach. The research can help in the development of the SCS method, deeper understanding of the catalytic properties of graphene-based materials, and their composites with metal nanograins. Also, we believe, the obtained NMs have the great practical potential as heterogeneous catalysts.

## Results and discussion

### XRD and Raman spectroscopy

**Figure 1:**
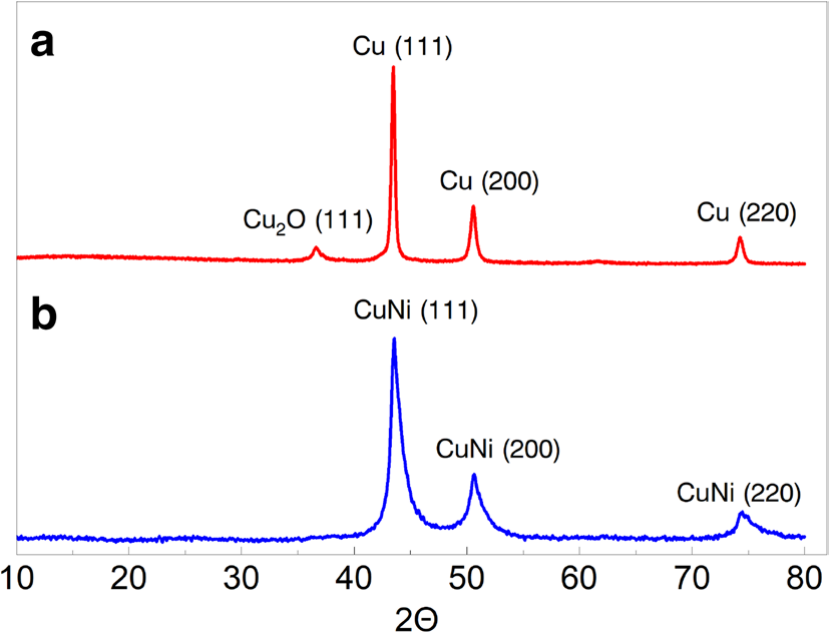
XRD patterns of (**a**) G@Cu and (**b**) G@CuNi samples.

**Figure 2:**
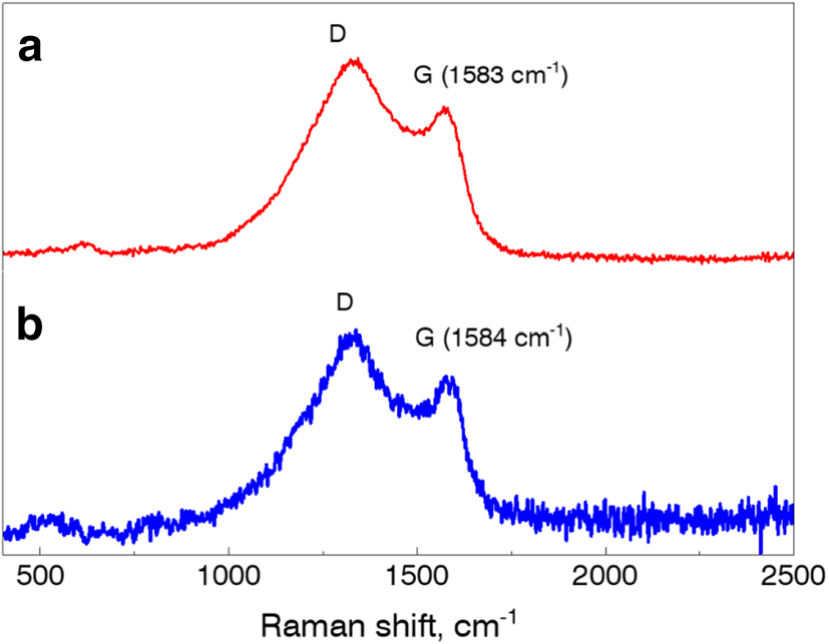
Raman spectrum of (**a**) G@Cu and (**b**) G@CuNi samples.

The results of XRD (Fig. 1) and Raman (Fig. 2) spectroscopy analysis of the samples show the powders are consist of two main components: metal/bimetallic crystals and scsG phase.

The Cu in G@Cu samples has a face-centered-cubic (fcc) crystal structure (Fm3m space group) with characteristic XRD peaks at 43.48° (111), 50.60° (200) and 74.30° (220), respectively, with the calculated crystallites size of 31 nm (Fig. 1a). The powder also contains minor Cu_2_O by-phase (~ 2.5 wt%), which is common for the Cu metal phase, obtained by SCS in the air using CA as a fuel^16^. The metal component of the G@CuNi samples is the single-phase bimetallic CuNi nanoalloy with a calculated crystallite size of 14 nm. The characteristic peaks at 43.56° (111), 50.75° (200), and 74.48° (220) indicate a distorted fcc (Fm3m) crystal structure of CuNi (Fig. 1b).

We suppose, the absence of the Cu- or Ni-based oxide phases in the bimetallic sample could be explained by the stabilizing effect of Ni atoms, which, in composition with Cu, tend to form bimetallic alloys, known to be more oxidation resistant than pure copper^17^. Moreover, the formation of complex bimetallic nanoparticles could prevent crystalline growth during the high-temperature SCS process. As it was shown in our previous works^16,18^, initial nanoparticles of individual metals could significantly grow even during fast SCS reaction. However, we suppose, intensive crystalline growth could be limited, or even prevented by the competing process of the formation of the bimetallic phase. In this case, energy, required for particle coalescence, partly goes to overcome the activation barrier for the formation of complex bimetallic phase with slightly distorted crystalline cell^19^.

There are several peaks in the Raman spectra of graphene structures which are characteristic. For instance, G-band (∼1580 cm^−1^) is a tangential stretching (E2g) mode and corresponds to the sp2-hybridized hexagonal lattice of a highly oriented pyrolytic graphite. D-band at around ∼1350 cm^−1^ originates from the disorder in the sp2—hybridized carbon atoms in hexagonal rings^20,21^. The D-band is highly dispersive in the 2D G structure and any change in the electron structure of a π-band (i.e. functionalization, the appearance of defects, etc.) would change the intensity of the band due to change in the resonance value of the phonon moment^22^.

Figure 2a shows two major characteristic G peaks at 1322 cm^−1^ (D-band) and 1583 cm^−1^ (G-band) in the Raman spectrum of the G@Cu sample. The G-band peak position corresponds to the tri-layered G structure^23^. The calculated peaks intensity ratio I_D_/I_G_ of 1.254 indicates highly distorted or/and defected G structure, characteristic to scsG phase, described in detail earlier^14^. The D- and G-bands were also founded at 1327 cm^−1^ and 1584 cm^−1^ at the Raman spectrum of the G@CuNi sample (Fig. 2b). The G-band peak position and the I_D_/I_G_ ratio of 1.204 indicate a highly distorted and defected double-layered scsG structure^23^. The distorted structure of scsG could be explained by features of their formation mechanism during high-intensive fast SCS reactions. In this case, scsG film growth starts on metallic nanograins and then develops due-to a self-catalytic process, where products of CA decomposition play the role of carbon atoms source^14^. The combination of a local temperature gradient, gradients of concentration of reacting components, and products of reaction could create conditions for the start of growth of scsG from the multiple metallic clusters, simultaneously. This process results in the formation of a G, which is characterized by high defectiveness of its structure. We suppose as SCS reaction is fast, the reacting system has no enough energy for self-healing (recrystallization) of formed defected primary scsG. In its turn, the difference in the I_D_/I_G_ ratios of G@Cu and G@CuNi samples could be explained by the difference in the mechanisms of scsG formation on Cu and CuNi. Nickel has a higher than Cu value of carbon solubility, which is available for the formation of initial scsG on the surface of the bimetallic nanoparticles. We suppose, in this case, a higher amount of available carbon atoms could lead to a slightly better quality of initial scsG, which could affect the quality of scsG film overall.

The additional analysis of XRD patterns of the powders after the catalytic measurements (Supplementary Information Fig S1) shows that a significant amount of CuO crystal phase forms in G@Cu as a result of Cu nanograins oxidation by oxygen species during the CO conversion process. On the other hand, only traces of (CuNi)_x_O_y_ crystal phase were detected in the G@CuNi samples, used in catalysis.

### TEM characterization

**Figure 3:**
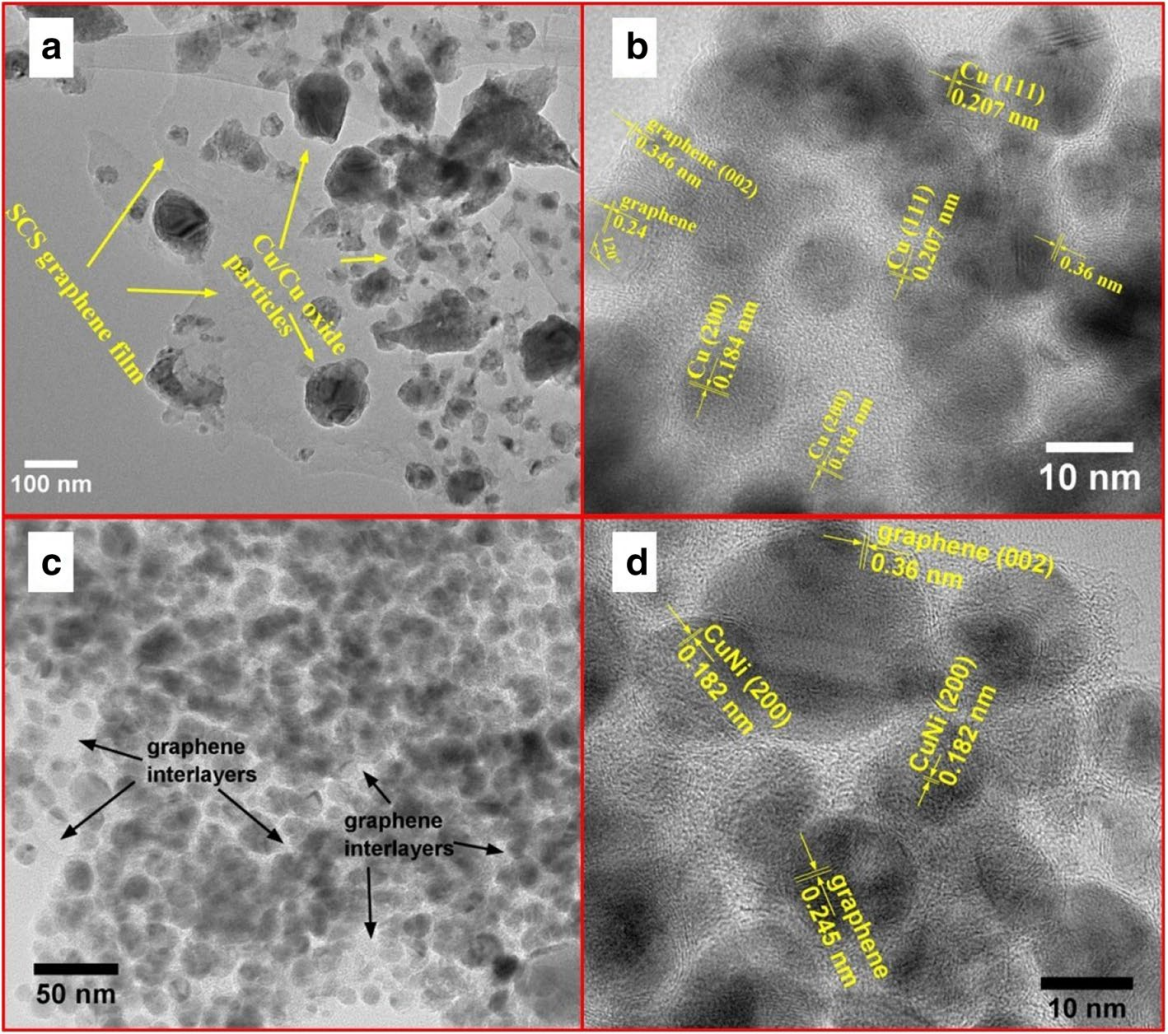
TEM images of (**a**, **b**) G@Cu and (**c**, **d**) G@CuNi powders.

The TEM images of the G@Cu and G@CuNi powders show features of the samples’ structure and morphology (Fig. 3).

The scsG forms a large-area film, covering Cu grains, which are mostly separated from each other by the scsG buffer layers. There are two types of grains of about 8–15 nm in diameter with d-spacing values of 0.207 nm and 0.184 nm, which correspond to (111) and (200) oriented Cu crystals (Fig. 3a,b). The scsG matrix is characterized by a d-spacing value of about 0.36 nm in (002) crystallographic direction, which is a thickness of a single graphene layer, by the angle of invariant crystallographical rotation of individual crystal cell of 120°, and typical C–C atoms distance in a honeycomb of about 0.24 nm.

The G@CuNi powder is a composite of 5–25 nm CuNi crystal grains and scsG (Fig. 3c,d). The scsG is presented in two forms: large-area film, covering CuNi bimetallic grains, and carbon multilayers with a single-layer thickness of 0.36 nm around bimetallic grains. The difference in forms of scsG in Cu and CuNi containing samples is caused by the features in mechanisms of formation of primary scsG, which is mainly related to a different carbon solubility values in Cu and CuNi grains. Further scsG film growth is related to the self-catalytic effect described earlier^24^.

### XPS characterization

**Figure 4:**
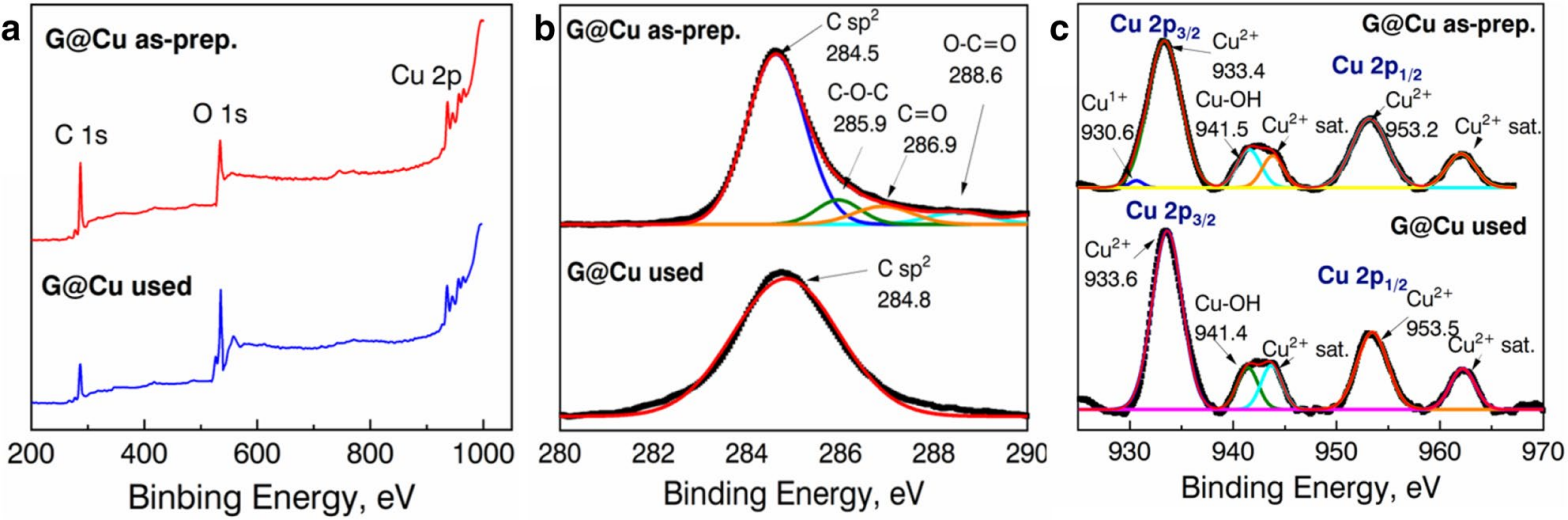
Comparative XPS analysis of (**a**) as-prepared and used in catalysis powders of the G@Cu sample, and enlarged view on separate peaks around (**b**) 280–290 eV (C 1s) and (**c**) 925–970 eV (Cu 2p).

**Figure 5:**
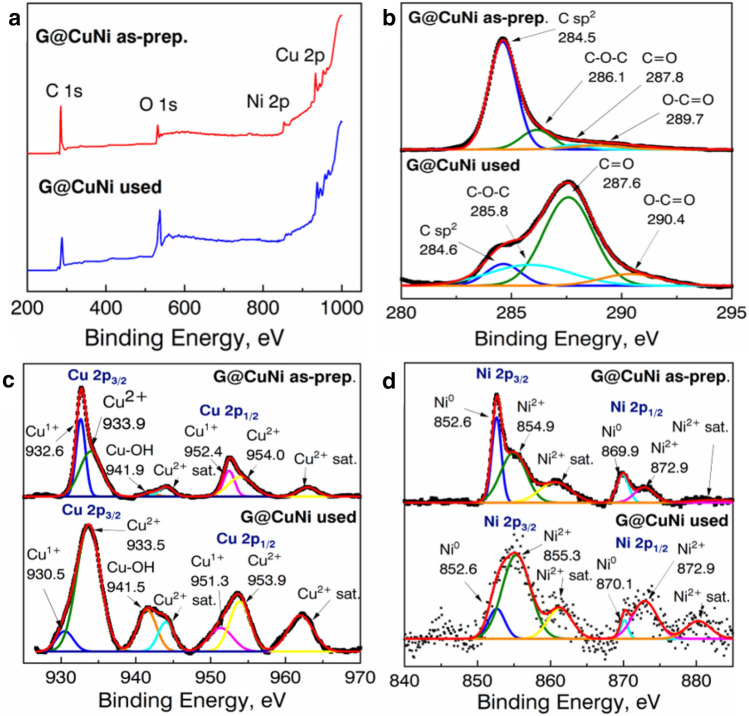
Comparative XPS analysis of (**a**) as-prepared and used in catalysis powders of the G@CuNi sample, and enlarged view on separate peaks around (**b**) 280–295 eV (C 1s), (**c**) 925–970 eV (Cu 2p), and (**d**) 840–885 eV (Ni 2p).

The results of comparative XPS analysis of as-prepared and tested in CO oxidation samples are shown in Figs. 4 and 5.

From the data in Fig. 4a it could be seen there are three characteristic peaks with binding energy values to C 1s, O 1s, and Cu 2p spectra, with elements’ concentration of 79.52 at.% 15.91 at.% and 4.57 at.% for as prepared powder, and 42.54 at.%, 55.63 at.% and 1.83 at.% for used powder, respectively. The elements ratios in both samples indicate the Cu nanograins are oxidized and covered with the scsG film, which corresponds with the TEM observation. The deconvolution of the C 1s spectra (Fig. 4b) of the as-prepared sample shows there are 4 peaks, characteristic for G structures: C–C sp^2^ band (284.5 eV), C–O–C (285.9 eV), C=O (286.9 eV) and O–C=O (288.6 eV)^25^. On the other hand, only one peak at 284.8 eV corresponds to the C–C sp^2^ bond was found in the XPS spectrum of the tested in the CO conversion sample.

The deconvolution of the Cu 2p spectrum (Fig. 4c) of the as-prepared G@Cu samples shows the presence of 4 main peaks at 930.6 eV, 933.4 eV, 941.5 eV, and 953.2 eV, and two Cu^2+^ satellite peaks, indicating the mostly 2 + oxidized surface of Cu nanograins^26^. In the spectrum of the used G@Cu sample, only peaks correspond to Cu^2+^ were found. The absence of a 930.6 eV peak, indicating full oxidation of the residual Cu^1+^ species by oxygen atoms, involved in CO conversion.

Four XPS spectra, correspond to C 1s, O 1s, Cu 2p, and Ni 2p were found in both G@CuNi samples (Fig. 5a). The calculated concentration of elements on the surface of the powders is 84.46 at.%, 10.34 at.%, 3.31 at.% and 1.89 at.% for as-prepared sample, and 59.81 at.%, 36.72 at.%, 3.16 at.% and 0.31 at.% for used sample, respectively. The high C concentration indicates the scsG layers, covering metallic grains.

The deconvolution of the C 1s spectra of the G@CuNi samples shows (Fig. 5b) both the as-prepared and used in catalysis samples have four characteristic peaks of the G structure as in the as-prepared G@Cu sample. However, peaks in the XPS spectrum of the used G@CuNi sample are characterized by a significantly lower intensity of peak corresponds to C–C sp^2^, and C=O peak is the most intensive. This may indicate the high value of absorbed oxygen species by the scsG structure, which is characteristic for GO type materials^25^. We suppose the scsG structure partly oxidizes and transforms into GO. The analysis of the deconvoluted Cu 2p XPS spectrum of the as-prepared G@CuNi sample shows (Fig. 5c) the presence of high-intensive peaks of Cu^+1^ at 932.6 eV and 952.4 eV along with peaks correspond to Cu^2+^. The Cu^1+^ peaks were also found at the spectrum of the used G@CuNi powder. Six peaks were founded at the deconvoluted XPS Ni 2p spectra of the G@CuNi samples (Fig. 5d). The 852.5 eV and 869.9 eV peaks in the XPS spectra of the as-prepared sample and 852.6 eV and 870.1 eV in the spectra of the used one indicate the presence of metallic Ni. The other two main peaks at 854.9 eV and 872.9 eV for the as-prepared sample, and 855.3 eV and 872.9 eV for the used powder, as well as two satellite peaks, indicates Ni^2+^^27^.

We suppose the presence of metallic nickel, as well as Cu^+1^ bonds in samples after using them in CO oxidation process, confirms higher oxidation resistivity of bimetallic CuNi NPs in comparison with Cu and may influence samples' catalytic activity and stability.

### Surface area and pore characteristics

The results of BET measurements showed that the obtained structures are characterized by high values of the surface area of 117.1 m^2^/g (G@Cu) and 140.9 m^2^/g (G@CuNi). The pore diameter distribution and pore volume data (Supplementary Information Figs. S2 and S3) show, the calculated cumulative pore volume in pore diameters range 0–300 nm reaches 0.27 cm^3^/g and 0.23 cm^3^/g for G@Cu and G@CuNi, respectively. It is worth to notice, that in both cases more than a half pore volume is originating from the pores with diameters from 3.5 to 29.5 nm with a peak value at around 8 nm.

We suppose the higher surface area of the G@CuNi in comparison with the G@Cu could be explained by the formation of scsG films of higher quality with a fewer number of layers and pores, which is indirectly confirmed by the difference in the cumulative pore volume values.

Overall, the high values of the surface area are common for various G-metal nanocomposites and mostly obligated to features of G. In its turn, the formation of the highly porous structure of the G@Me nanomaterials is a characteristic feature of the SCS process, where a large volume of gases evaluates during combustion. The gases could mix reacting components in a local volume and transfer heat. We suppose this feature could also influence the scsG formation by determining self-catalytic scsG growth directions through changing local components concentration and heat gradient.

### Catalityc properties

The combination of metallic and scsG phases and features of morphology make the synthesized NMs promising candidates for application in catalysis. For this research, G@Cu and G@CuNi samples were examined in two types of heterogeneous catalytic processes: thermal oxidation of CO and photocatalytic decomposition of ATB and DB dyes in water.

#### CO oxidation

**Figure 6:**
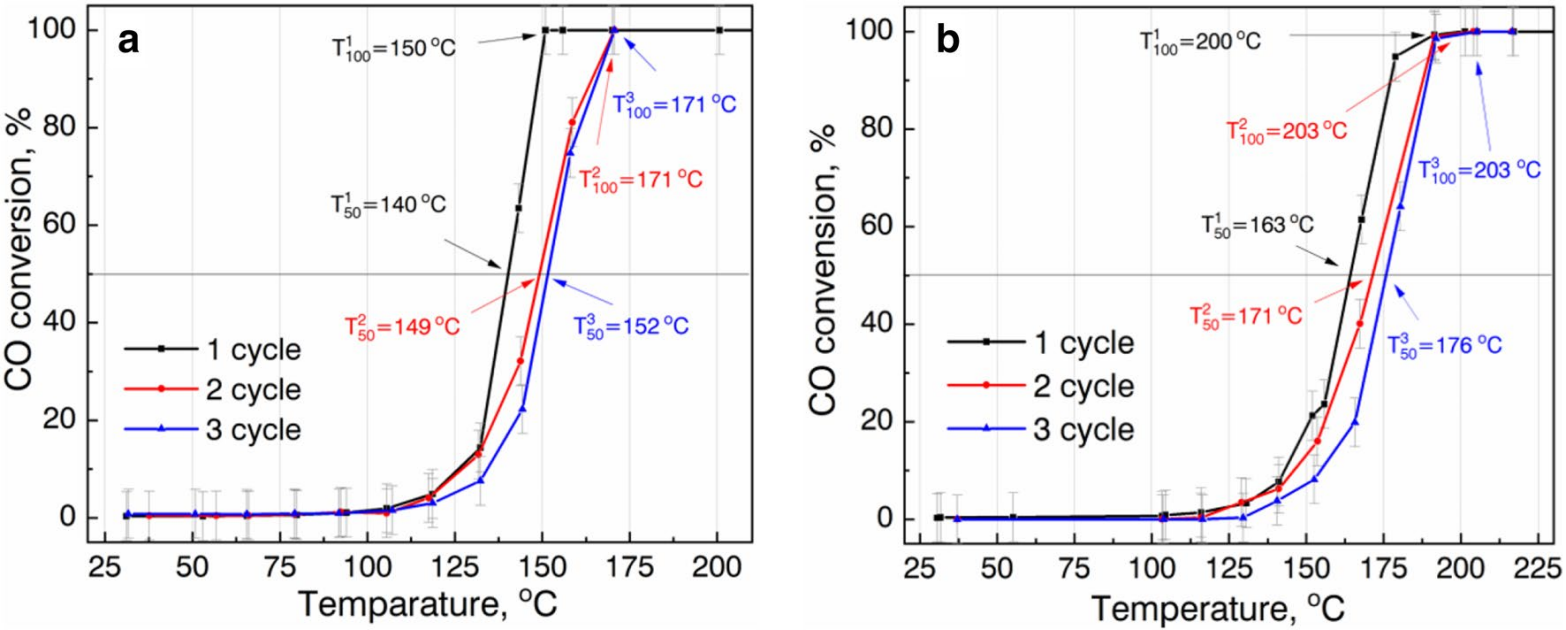
Catalytic activity for CO oxidation of (**a**) G@Cu and (**b**) G@CuNi nanopowders.

The results of the catalytic CO oxidation activity measurements for both experimental samples are presented in Fig. 6.

From the data, one can see, both samples have sigma-type shapes catalytic curves. At the first measurement cycle, there is a sharp increase of catalytic activity at 132 °C and 152 °C for G@Cu (Fig. 6a) and G@CuNi (Fig. 6b) samples, respectively. The G@Cu sample reaches 50% conversion at T_50_ = 140 °C, and 100% at T_100_ = 150 °C. The catalytic activity of the G@CuNi sample is lower, as its values of T_50_ and T_100_ of 163 °C and 200 °C are higher on 23 and 50 degrees, respectively.

The results of stability tests of catalysts (the second and the third cycles) show the T_50_ value of G@Cu catalysts shifts from 140 °C, at the first oxidation cycle, to 149 °C and 152 °C at the second and the third cycles (increase on 9 and 12 degrees), and the T_100_ value increases from 150 °C to 171 °C and 171 °C, respectively. The T_50_ values for the G@CuNi catalyst reach 171 °C and 176 °C at the second and the third cycles (increase on 8 and 13 degrees), respectively, as T_100_ value at the first oxidation cycle is almost the same as at the second and the third cycles (201 °C, 203 °C and 203 °C, respectively).

It is well known, the metal and oxide nanopowders^9,28^, as well as graphene-based materials with functional groups and defects of different nature, play one of the major roles in catalysis, by activating oxygen dissociation and electron transfer between gas molecules and catalysts surface^13,29^. Moreover, combining metal grains, containing thin oxide film on their surfaces, with scsG could create a p-p heterojunction with a very wide electron depletion layer^30,31^. We suppose, in this case, adsorbed CO molecules play the role of electron donors, forming positively charged radicals and easily interact with negatively charged oxygen radicals on the scsG-metal surface. Such a structure could decrease the activation energy of CO oxidation and promote conversion reaction at lower temperatures.

The difference in the catalytic activity of the samples could be explained by a difference in the properties of metal phases and, also, due to scsG-metal interaction and characteristics of the scsG in both NMs. As it was shown by Raman spectrum study (I_D_/I_G_ values), scsG, formed on Cu grains, is characterized by higher defectiveness of the structure in comparison with scsG on CuNi grains. Also, the higher T_100_ value for G@CuNi could indicate lower kinetic of thermal activated CO oxidation in the case of the G@CuNi sample in comparison with the G@Cu. The difference could be caused by a difference in the energy of the formation of nickel and copper oxides. It follows from the well known Sabatier principle that bond strength between oxygen and catalyst material should have intermediate value. Taking into account the oxide formation energies of well-optimized state-of-art Pt and Pd CO oxidation catalysts are − 133.9 and − 115.5 kJ/mol, respectively, the difference between the two studied systems can be explained by closer to the optimal value of Cu oxide formation (− 170.6 kJ/mol) in comparison with Ni oxide (− 239.7 kJ/mol) (values of formation enthalpies are taken from HSC Chemistry 5 software). Thus, the higher strength of the Ni–O bond leads to the increase of irreversibly bound oxygen on the surface of the G@CuNi catalyst which, in turn, resulted in the decrease of catalytic active site number.

The increase of values of the characteristic temperatures at the second and the third oxidation could also be explained by the poisoning of catalysts’ surfaces by the oxygen species during the conversion process. The results of the XPS (Figs. 4 and 5) and XRD (Supplementary Information Fig S1) studies of catalysts, used in the CO conversion, show both experimental samples are characterized by the appearance of metal oxide phases. Based on the comparative analysis of stability tests, XPS and XRD data, we suppose, the main factor influence decrease of catalytic activity of the samples is metal grains oxidation. It was shown, the Cu in G@Cu samples is characterized by a high degree of oxidation after using in catalysis, as CuNi nanograins are more stable with a significantly lower degree of oxidation. The formation of copper (II) oxide in the G@Cu sample during the first cycle leads to the deactivation of catalyst since Cu^+^ and Cu^0^ species play a crucial role in catalytic CO oxidation reaction^32,33^. On the other hand, the phase stabilization effect of Ni in the G@CuNi catalyst leads to the enhanced stability of the catalyst.

The role of the scsG in the catalytic activity of the samples is not clear yet. The presence of the scsG films significantly increases the surface area of the catalysts and help absorb gas molecules on their surface. However, as T_100_ is almost the same for all three cycles for the G@CuNi sample, we suppose the oxidation of the scsG doesn’t affect catalytic activity significantly. It could mean, the main role of the scsG films is to absorb gas molecules on their surface, and the main catalytic role belongs to metal grains. At the same time, as it was shown by the XPS study, the surface of the scsG of the tested in catalysis G@Cu sample was completely reduced and no absorbed oxygen species were found. The nature of the process is not clear. We could speculate, that absorbed on the scsG surface groups are also involved in CO oxidation. And their reduction at the first cycle could also negatively influence the catalytic activity of the G@Cu at the second and the third cycles.

Overall, we can conclude, both NMs are among the best catalysts for thermal activated CO oxidation.

#### Dye degradation

**Figure 7:**
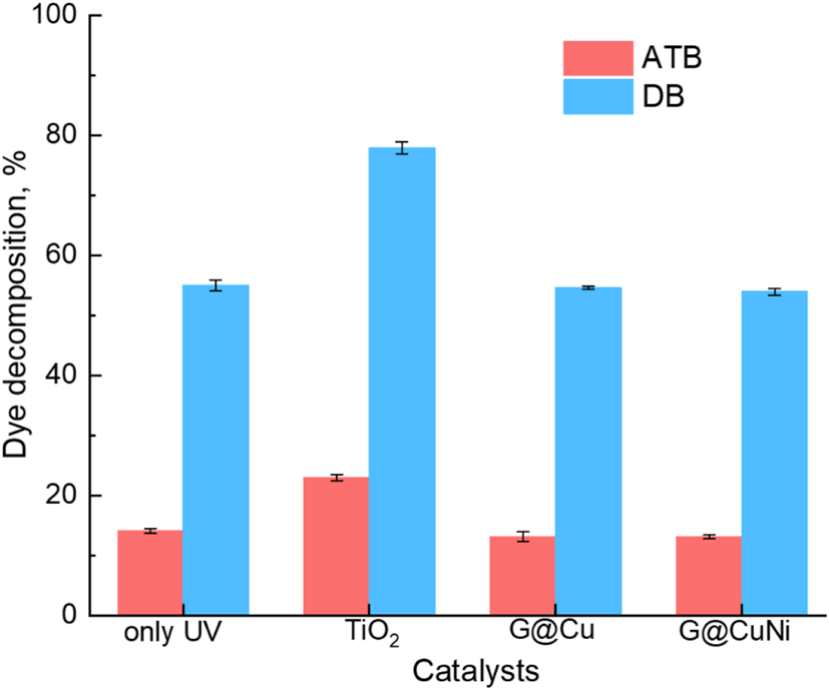
Photocatalytic degradation of ATB dye aqua solutions by UV and UV with catalysts.

The results of a comparative assessment of photocatalytic activity were studied under the UV degradation of ATB and DB dyes comparing with the TiO_2_ powder as a standard (Fig. 7).

It could be seen, the decomposition under the only UV resulted in a 14.1% and 55.0% reduction of ATB and DB concentration, respectively. This indicates ATB high stability and was described before for other dyes^34,35^. Photocatalytic decomposition using TiO_2_ under UV gave a concentration decrease of ATB on 23.0%, and of DB on 77.9%, showing clear activation of the decomposition process by catalysts. In the case of using G@Cu and G@CuNi powders, no significant change in the concentration of the dyes in comparison with UV treatment was found. The same zero effect was observed in the dyes sorption on the surface of materials exposed under UV in the solution for 24 h.

We suppose the result could be explained by the characteristic of UV, in which the radiation spectrum is not in the optimal range for activation of G@Cu and G@CuNi powders. Moreover, poorly wetted graphene-based materials could block massive dye molecules from penetration to metal surface and prevent them from decomposition.

## Conclusion

In summary, scsG-Cu and scsG-CuNi composites NMs were studied in heterogeneous catalytic processes of CO oxidation, ATB, and DB dyes decomposition. We found that both NMs are among the best catalysts for CO oxidation with a temperature of 100% conversion of 150 °C and 200 °C, due to features of their structure and scsG-metal interaction. At the same time, the samples were found catalytically inactive in the dyes decomposition process. The results of the research are the step for the study of newly obtained scsG and could be useful for the development of effective catalysts for various gas conversion processes.

## Methods

### Synthesis

The experimental samples were obtained by the SCS method as described in our previous article^14^. Briefly, in typical experiments, 12.23 g of citric acid (CA, Chimmed, 98.9%; Russia) and 3.78 g of Cu(NO_3_)_2_·3H_2_O (Chimmed, 98%; Russia) (G@Cu sample), or 12.09 g of CA, 1.89 g of Cu(NO_3_)_2_·3H_2_O and 2.47 g of Ni(NO_3_)_2_·6H_2_O (Chimmed, 99%; Russia) (G@CuNi sample) were dissolved in hot distillate water. The fuel-to-oxidizer ratio φ for all samples was kept equal to 5. The experimental solutions were fast dried at 120 °C and then burned in a muffle furnace at 600 °C. The fast drying allows obtaining precursor in the form of a porous easy combustible foam. The temperature of the furnace for SCS reaction initiation was chosen based on an analysis of the result of our previous study^14,36,37^. It was shown, the decomposition of the precursor as a result of the combustion reaction mostly finishes up to ∼ 500 °C. In this case, a slightly higher temperature of 600 °C guarantees complete decomposition of initial components and, at the same time, it is low enough to prevent oxidation of the metallic phase and burnout of the graphene.

### Materials characterization

The phase composition and structure of the obtained NMs were characterized by XRD with Cu Kα radiation (Rigaku Ultima IV diffractometer, Japan). The size of the crystallites was calculated by the Scherrer equation using the main (111) peak. Raman spectrum study was carried out under a 780 nm laser using a 20′ Senterra Raman microscope (Bruker, Germany). Transmission electron microscopy (TEM) images were taken on JEM-2100, 200 kV LaB6 instrument (JEOL, Japan) using a carbon-coated copper grid for samples’ preparation. The surface area and pore characteristics of the samples were measured using 3Flex analyzer (Micromeritics, Germany). Before measurements, 0.5 g of powder of each sample was degassed for 12 h under vacuum (0.05 mbar) at 300 °C. Surface area and pore characteristics were calculated using 3Flex software by the BET and DFT modeling, respectively.

The XPS spectra were recorded using electron spectrometer PHOIBOS 150 MCD-9 (SPECS, Germany), equipped with an X-ray tube (magnesium anode, hν = 1253.6 eV). The vacuum in the spectrometer chamber during the experiment was kept less than 3·10^−8^ Pa. The source power was 225 W and the spectra were recorded in the constant transmission energy mode (40 eV for full spectra and 10 eV for individual lines). The full spectra were recorded with a step of 1.00 eV and individual lines, with a step of 0.05 eV.

### Catalytic properties study

The catalytic activity of samples for the CO oxidation reaction was evaluated in a continuous flow reactor at atmospheric pressure. 50 mg of sample was placed inside a quartz tube with 4 mm inner diameter. Before the analysis samples were in-situ activated in a flow of 10% H_2_/He (total flow rate of 36 mL/min) at 350 °C for 1 h. The furnace was then cooled down to room temperature and the flow was changed to a gas mixture of 5.6% CO and 11.1% O_2_ in He balance (total flow rate 36 ml/min, GHSV = 43,200 ml g^−1^ h^−1^). The test was then performed in a temperature range between 50 and 250 °C. The data were collected after the catalyst was stabilized for 30 min at each temperature. The effluent gas composition was determined using quadrupole mass-spectrometer (Thermostar GSD 320, Pfeiffer Vacuum, Germany) and CO conversion was calculated according to Eq. (1)^38^:

1 $$X=1- \frac{{f}_{CO}^{f}}{{f}_{CO}^{i}};$$

where ${f}_{CO}^{f}$—CO gas flow rate after the catalyst bed; ${f}_{CO}^{i}$—initial CO gas flow rate.

The second and the third measurement cycles for stability tests were performed successively using the same load of the experimental powders without an additional reduction in hydrogen after the previous cycle.

An aqueous solution of an acid telon blue (ATB) and direct blue (DB) dyes with a concentration of 10 mg/L and a volume of 50 ml was used to analyze the catalytic activity of the samples in the degradation of organic substances process. TiO_2_ powder was used as a standard catalyst. The chosen dose of the catalysts used in the experiment was 500 mg/L. Catalytic treatment was carried out using a home-made set-up with an ultraviolet DRT-400 mercury-quartz lamp (emitted radiation wavelength is 240–320 nm and radiant energy power is 36 W) for 45 min. The solutions with catalysts were constantly stirred. The dye concentration was measured by the spectrophotometric method using Spectrophotometer PE-5400UV (Ecroskhim, Russia). To remove particulate before analysis, all samples were centrifuged at 3000 min^−1^ for 3 min.

## Supplementary information

Supplementary Figures.

## Data Availability

The datasets generated during and/or analyzed during the current study are available from the corresponding author on reasonable request.
