# Supplementary material for: CO oxidation and organic dyes degradation over graphene–Cu and graphene–CuNi catalysts obtained by solution combustion synthesis
Source: Sci Rep. 2020 Sep 30;10:16104. doi: 10.1038/s41598-020-72872-0 (PMC7527964; doi:10.1038/s41598-020-72872-0)
Supplement: Supplementary file 1 — Supplementary Figures. [file 41598_2020_72872_MOESM1_ESM.docx]

**Supplementary information**

**For**

**CO oxidation and organic dyes degradation over graphene-Cu and graphene-CuNi catalysts obtained by solution combustion synthesis**

Alexander Khort^a,b,*^ , Valentin Romanovski^b,c^ Denis Leybo^b^, Dmitry Moskovskikh^b^

^a^KTH Royal Institute of Technology, Stockholm 10044, Sweden

^b^National University of Science and Technology “MISIS”, Moscow 119049, Russia

^c^Institute of General and Inorganic Chemistry, National Academy of Sciences of Belarus, Minsk 220072, Belarus

*E-mail: khort@kth.se


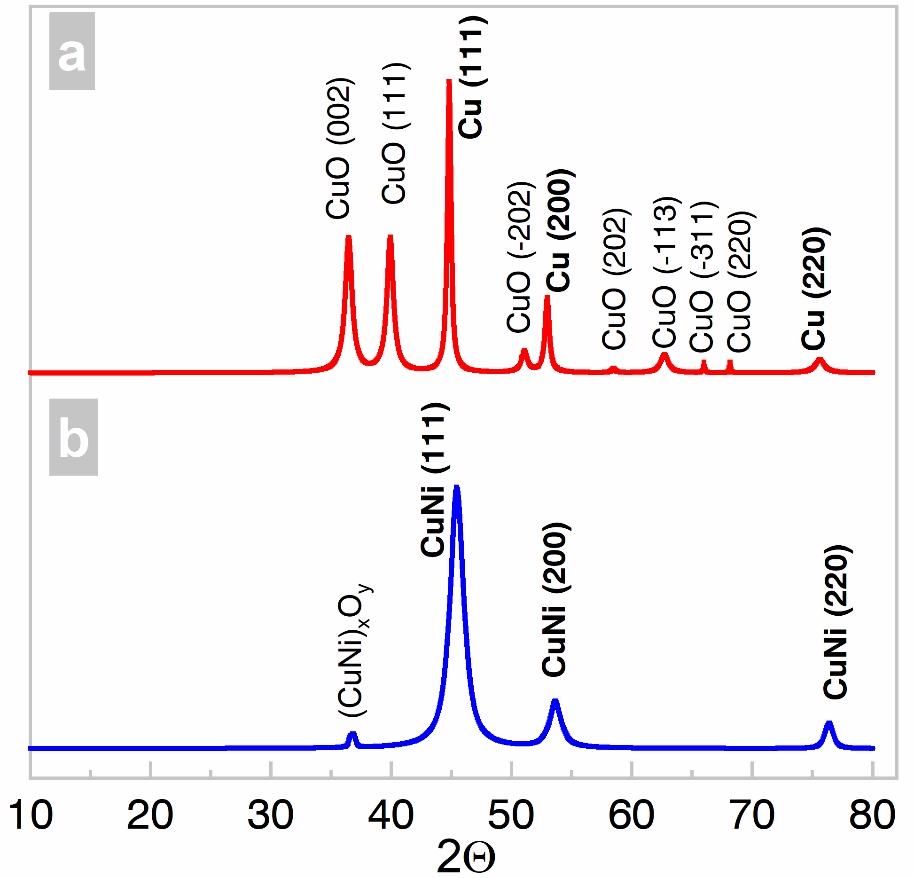


Figure S1. XRD patterns of (a) G@Cu and (b) G@CuNi samples, used in catalytic CO conversion.

Measured pore diameter distribution and pore volume in samples G@Cu and G@CuNi are shown in Figs S2 and S3, respectively.


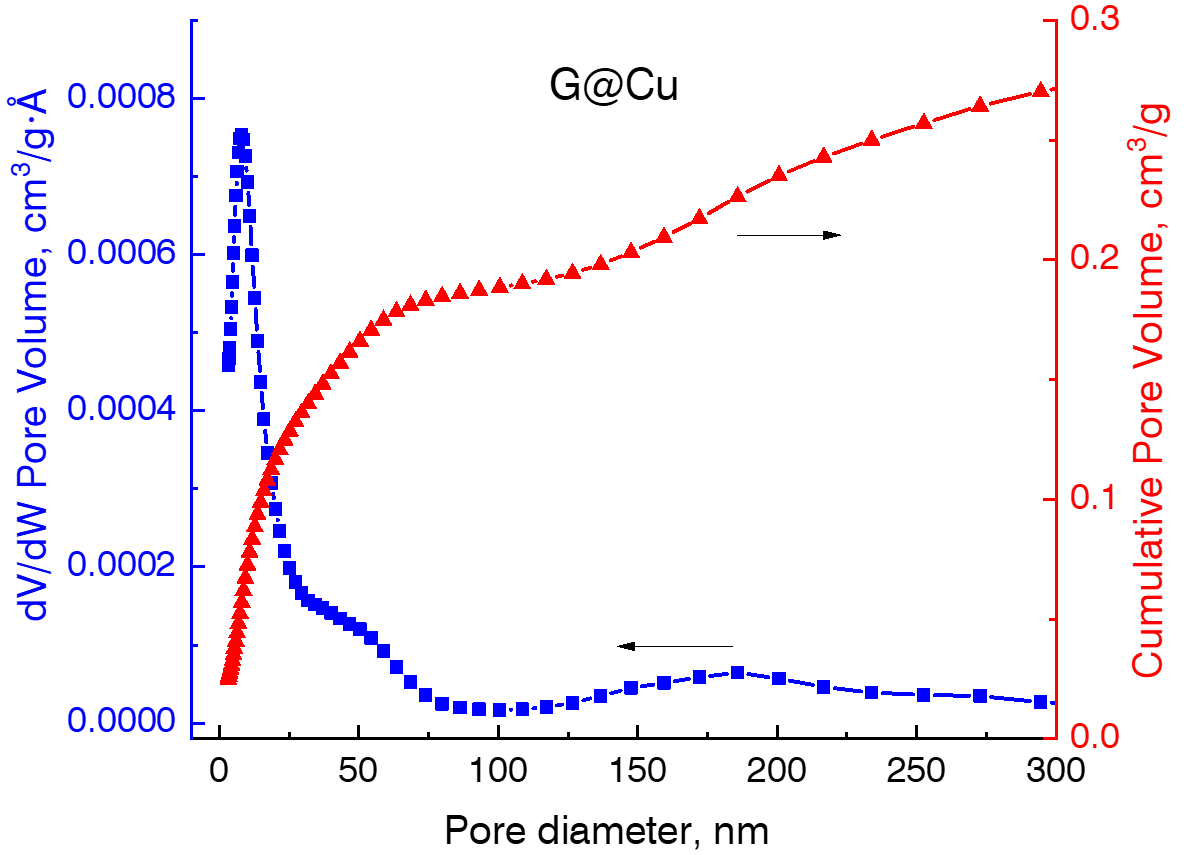


Figure S2. Pore diameter distribution and pore volume in G@Cu sample


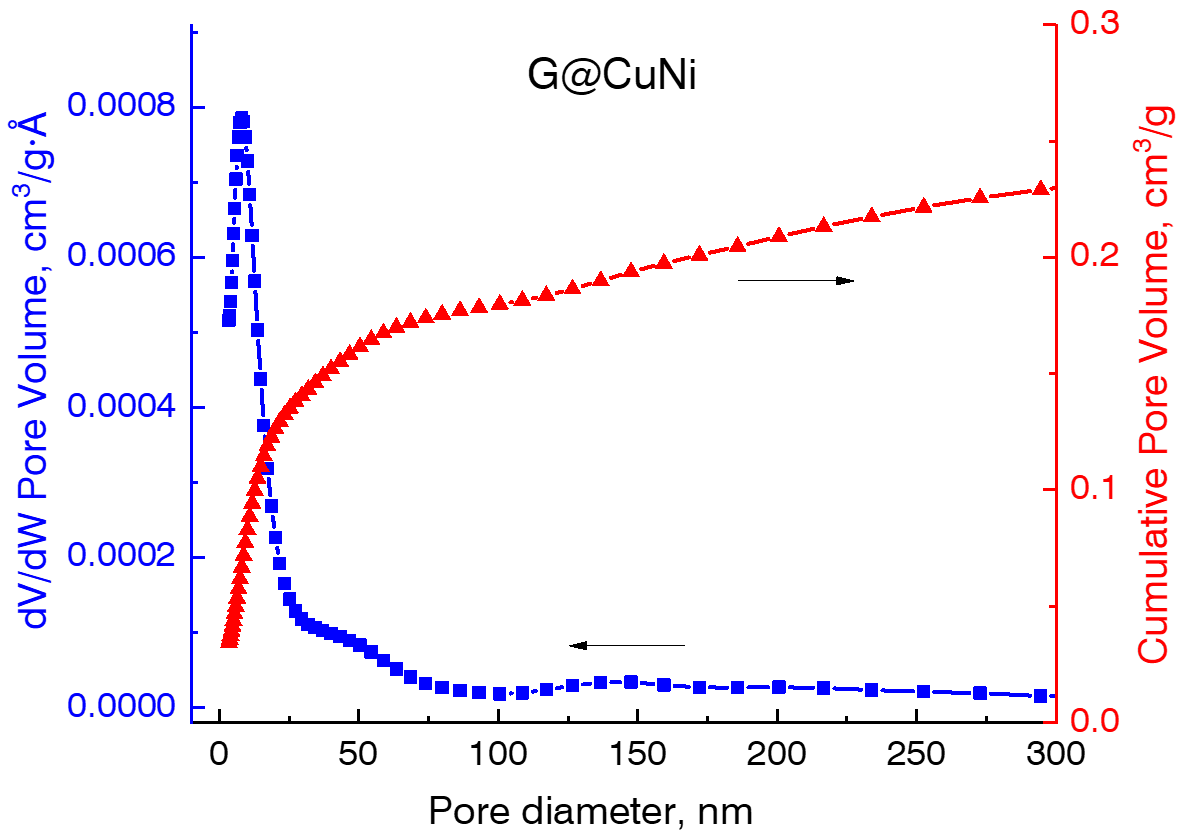


Figure S3. Pore diameter distribution and pore volume in G@CuNi sample
